# Age-Related Shift in Cardiac and Metabolic Phenotyping Linked to Inflammatory Cytokines and Antioxidant Status in Mice

**DOI:** 10.3390/ijms242115841

**Published:** 2023-10-31

**Authors:** Ryeonshi Kang, Charlotte Laborde, Lesia Savchenko, Audrey Swiader, Nathalie Pizzinat, Dimitri Marsal, Yannis Sainte-Marie, Frederic Boal, Helene Tronchere, Jerome Roncalli, Oksana Kunduzova

**Affiliations:** 1 National Institute of Health and Medical Research (INSERM) U1297, CEDEX 4, 31432 Toulouse, France; ryeonshi.kang@inserm.fr (R.K.); lesia.savchenko@inserm.fr (L.S.); audrey.swiader@inserm.fr (A.S.); nathalie.pizzinat@inserm.fr (N.P.); dimitri.marsal@inserm.fr (D.M.); yannis.sainte-marie@inserm.fr (Y.S.-M.); frederic.boal@inserm.fr (F.B.); helene.tronchere@inserm.fr (H.T.); roncalli.j@chu-toulouse.fr (J.R.); 2 University of Toulouse III, CEDEX 9, 31062 Toulouse, France; laborde.c@chu-toulouse.fr; 3 Department of Internal Medicine, Poltava State Medical University, 23 Shevchenko, 36000 Poltava, Ukraine; 4 Department of Cardiology, University Hospital of Toulouse, CEDEX 9, 31400 Toulouse, France

**Keywords:** aging, cardiac function, metabolic status, antioxidant, inflammation and resistin

## Abstract

Age-related alterations in cardiac function, metabolic, inflammatory and antioxidant profiles are associated with an increased risk of cardiovascular mortality and morbidity. Here, we examined cardiac and metabolic phenotypes in relation to inflammatory status and antioxidant capacity in young, middle-aged and old mice. Real-time reverse transcription–polymerase chain reactions were performed on myocardium and immunoassays on plasma. Left ventricular (LV) structure and function were assessed by echocardiography using high-frequency ultrasound. Middle-aged mice exhibited an altered metabolic profile and antioxidant capacity compared to young mice, whereas myocardial expression of inflammatory factors (TNFα, IL1β, IL6 and IL10) remained unchanged. In contrast, old mice exhibited increased expression of inflammatory cytokines and plasma levels of resistin compared to young and middle-aged mice (*p* < 0.05). The pro-inflammatory signature of aged hearts was associated with alterations in glutathione redox homeostasis and elevated contents of 4-hydroxynonenal (4-HNE), a marker of lipid peroxidation and oxidative stress. Furthermore, echocardiographic parameters of LV systolic and diastolic functions were significantly altered in old mice compared to young mice. Taken together, these findings suggest age-related shifts in cardiac phenotype encompass the spectrum of metabo-inflammatory abnormalities and altered redox homeostasis.

## 1. Introduction

Aging is an inevitable event of the lifecycle associated with an elevated risk of heart failure [1]. Age-related progressive decline in cardiac function differs across species, organs and tissues [2,3,4]. As the global population faces a progressive shift towards a higher median age, decoding the pathophysiological picture of an aged heart has become of paramount importance to the preservation of cardiac function. Among the various diseases encountered during aging, heart disease is prevalent among older populations, affecting their healthy lifespan and quality of life. Cardiac aging is driven by a finite number of inter-connected mechanisms that ultimately lead to the emergence of specific phenotypes, calibrating increased susceptibility to multiple chronic manifestations and death. Among the many critical aspects of age-related myocardial remodeling processes, oxidative stress inflammation and functional reprogramming are by far the most relevant [5]. The prevalence of heart disease is common in both humans and animals, manifesting typically as progressive loss in cardiac function, cytokine/adipokine dysfunctions and antioxidant reserve [6,7,8]. In mice, cardiac aging closely recapitulates human heart aging, which involves hypertrophy, diastolic dysfunction, reduced functional reserve and adaptive capacity to stress [2,7]. Echocardiographic imaging performed on a mouse longevity cohort demonstrated distinct age-dependent linear trends in increased left ventricular mass index (LVMI) and left atrial dimension, reduction in fractional shortening (FS) and diastolic function (Ea/Aa), as well as worsening of myocardial performance index [3]. Similar cardiac phenotypes have also been observed in aging of C57BL/6 mice [9], suggesting that age-mediated cardiac alterations observed in mouse and human are comparable. However, it is still unclear how these imaging modalities can be used to explore aging with mice models. Age-dominated dynamic changes in ventricular function may be monitored using non-invasive cardiac imaging. Echocardiographic measurements are important in the detection of cardiac abnormalities and in defining personalized aetiology of heart failure by providing well-defined parameters on ventricular structure and function [2,10]. In preclinical research, echocardiographic imaging of ventricular function and structure is a powerful approach to cardiac phenotyping, beneficial in the validation and characterization of disease progression, drug screens as well as exploration of heart damage and recovery [2,7,10]. In contrast to humans, mice have a naturally short lifespan, which makes a mouse a particularly interesting model for preclinical exploration of cardiac aging. Recent advances in imaging technology, with improved resolution, have made echocardiographic imaging in mice a universal tool for dissecting the physiological and pathophysiological alterations linked to age-associated phenotypes [11]. Yet, there is little comprehensive study related to age-associated cascade of cardiovascular events calibrating systolic and diastolic functions in relation to metabolic status and antioxidant capacity. Aging is characterized by excessive generation of high levels of reactive oxygen species (ROS) and defects in antioxidant capacity that can affect the heart’s physiological function [12,13]. The imbalance between antioxidant defense system and ROS production is the principal cause of oxidative damage, culminating in progressive loss of structural and functional parameters of the myocardium [13,14,15]. At the cellular level, the enhanced production of ROS leads to peroxidation of lipids, oxidation of proteins, damage to nucleic acids and activation of programmed cell death [14]. The first line of defense against ROS are catalase, superoxide dismutase (SOD) and glutathione peroxidase which play the capital roles in protecting the heart from oxidative stress and endorsing delayed cardiac aging [10,16]. Several studies suggest that catalase and SOD may alleviate cardiac disease and aging in a mouse model [17,18,19], suggesting a principal role of these enzymes in age-mediated myocardial remodeling. The purpose of the present study was to characterize age-associated progressive changes in cardiac phenotype in relation to metabolic, antioxidant and inflammatory status in mice.

## 2. Results

### 2.1. Age-Dependent Cardiac Phenotype in C57BL/6 Mice

To characterize age-dependent cardiac phenotypes in a cohort of male C57BL/6 mice, we compared physiological, metabolic and echocardiographic parameters in three age groups: young (3 months old), middle-aged (12 months old) and old (24 months old). Metabo-physiological status was the first characteristic assessed due to its high human relevance as the established parameters of clinical outcomes (Table 1).

**Figure 1:**
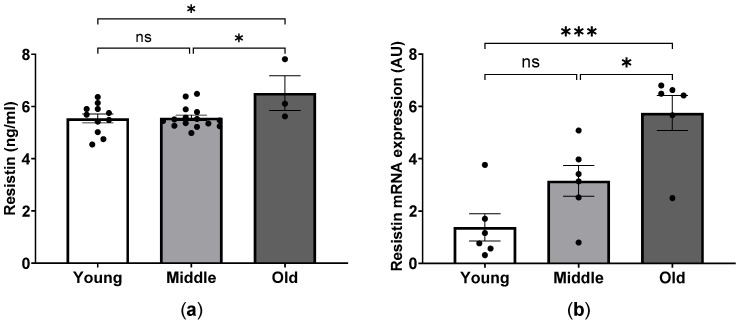
Age-dependent profile of resistin expression in mice. Plasma level (**a**) and quantitative RT-PCR analysis of mRNA expression (**b**) of resistin in young, middle-aged and old mice. * *p* < 0.05 and *** *p* < 0.001 between the indicated groups, ns, not significant, one-way ANOVA.

Group differences in body weight (BW) and whole-body fat were noted, with a significant increase in middle-aged mice and a decline thereafter in aged mice (Table 1). Compared to the young group, whole-body lean tissue was largely preserved in aged mice but significantly elevated in middle-aged animals. In addition, in aged mice, free body fluid was significantly increased as compared to young and middle-aged mice. Furthermore, age-associated alterations in metabolic status in mice were accompanied by increased mRNA and protein expression levels of adipokine resistin in mice (Figure 1).

### 2.2. Left Ventricular Dimensions in Young Middle-Aged and Aged Mice

Comparing mice according to age, absolute measurements of the left ventricle in the parasternal short-axis view demonstrated that the middle-aged group had significantly larger left ventricular anterior wall thickness (LVAW) and left ventricular posterior wall thickness (LVPW) versus the young group (Table 2).

**Figure 2:**
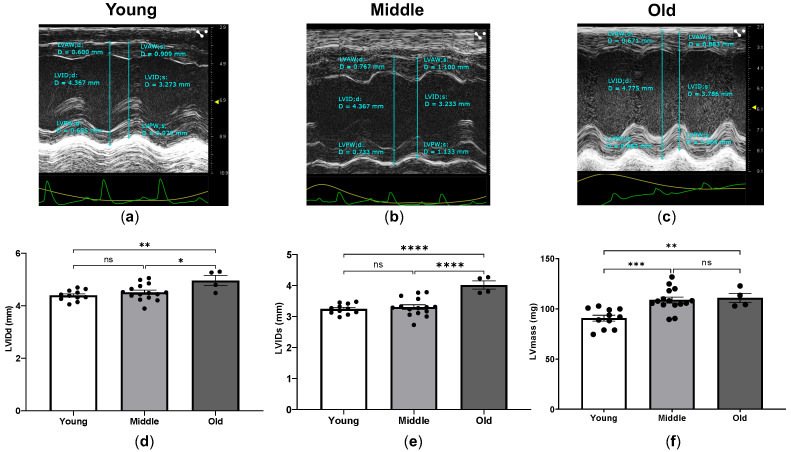
Parasternal short-axis M-mode images and age-associated comparisons of the left ventricular dimensions. Representative M-mode images of the parasternal short-axis view with measurements of the left ventricular dimensions in young (**a**), middle-aged (**b**) and old mice (**c**). Measurements of the left ventricular internal diameter in diastole (LVIDd) (**d**), left ventricular internal diameter in systole (LVIDs) (**e**), and left ventricular mass (**f**) in young, middle-aged and old mice. The echocardiographic examinations were performed and analyzed using the Vevo 2100 Imaging System (VisualSonics, Toronto, ON, Canada). * *p* < 0.05, ** *p* < 0.01, *** *p* < 0.001, and **** *p* < 0.0001 between the indicated groups, ns, not significant, one-way ANOVA.

As shown in Figure 2a–e, the left ventricular internal diameter (LVID) at diastole and systole measured from M-mode images revealed significant differences between young, middle-aged and old mice.

The LV mass was markedly increased in middle-aged mice compared to young mice and was subsequently maintained in old mice (Figure 2f).

### 2.3. The Echocardiographic Systolic Function in Young, Middle-Aged and Aged Mice

**Figure 3:**
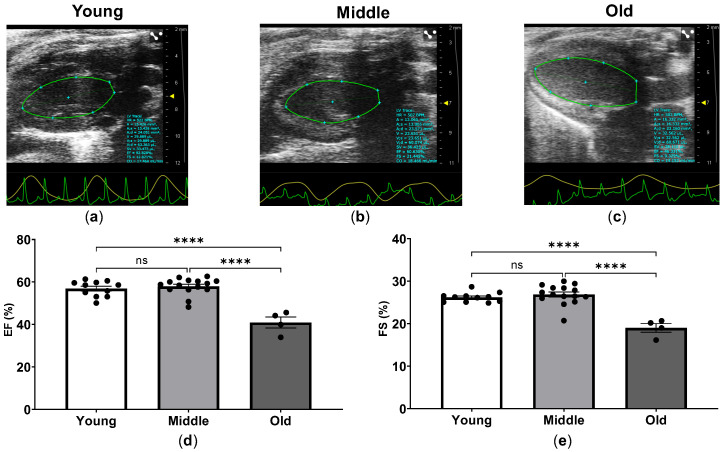
Parasternal long-axis images and LV systolic function in young, middle-aged and old mice. Representative images from parasternal long-axis view with the left ventricular tracing in systole (green) in young (**a**), middle-aged (**b**), and old mice (**c**). Measurements of the left ventricular ejection fraction (EF) (**d**) and left ventricular fractional shortening (FS) (**e**) in young, middle-aged and old mice. The echocardiographic examinations were performed and analyzed using the Vevo 2100 Imaging System (VisualSonics, Toronto, ON, Canada). **** *p* < 0.0001 between the indicated groups, ns, not significant, one-way ANOVA.

In order to evaluate LV systolic function in young, middle-aged and old mice, we analyzed ejection fraction (EF) in the parasternal long axis view and FS in the parasternal short axis view of the heart. As shown in Figure 3, the assessment of LV systolic function in mice revealed age-dependent decline of myocardial function. The mean EF was 56.87% for young, 57.92% for middle-aged, and 40.93% for old mice (Figure 3).

Both EF and FS were preserved in the middle-aged mice compared to the young mice, whereas declines in EF and FS were found in the old mice as compared to the young and middle-aged mice (Figure 3d,e). Measurements of stroke volume (SV) demonstrated a similar profile of changes with preservation of SV in middle-aged mice compared to young mice and then progressive decline in the old group. In contrast to EF, FS and SV, there was no significant difference in cardiac output (CO) between young and old mice (Table 2). There were no significant differences in heart rate between the three groups (Table 2).

### 2.4. The Echocardiographic Diastolic Function in Young, Middle-Aged and Aged Mice

**Figure 4:**
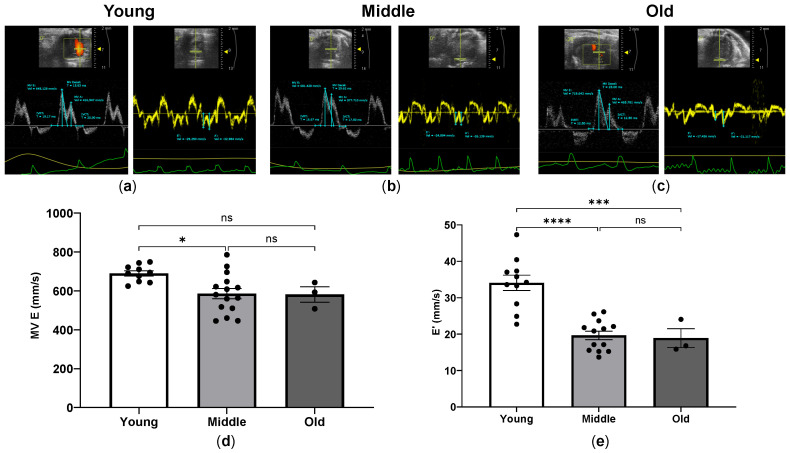
Pulse Doppler waveform of mitral inflow velocity and LV diastolic function in young, middle-aged and old mice. Representative images of pulse Doppler waveforms of mitral inflow velocity (white wave) and tissue Doppler imaging (yellow wave) in young (**a**), middle-aged (**b**), and old mice (**c**). Measurements of mitral valve inflow E wave (MV E) (**d**) and tissue Doppler-derived septal mitral annular E’ wave (E’) (**e**) in young, middle-aged and old mice. The echocardiographic examinations were performed and analyzed using the Vevo 2100 Imaging System (VisualSonics, Toronto, ON, Canada). * *p* < 0.05, *** *p* < 0.001, and **** *p* < 0.0001 between the indicated groups, ns, not significant, one-way ANOVA.

To assess the LV diastolic function in young, middle-aged and old mice, we obtained the mitral valve (MV) inflow velocity and tissue Doppler imaging from the apical four-chamber views. The representative apical four-chamber view images are presented, and MV E from MV inflow imaging and E’ from tissue Doppler imaging in young, middle-aged and old mice are shown in Figure 4.

The mean velocity of MV E was 690.0 mm/s for the young, 587.7 mm/s for middle, and 581.2 mm/s for old mice. The mean velocity of E’ in young, middle-aged, and old mice was 34.09 mm/s, 19.65 mm/s, and 18.91 mm/s, respectively. The velocity of MV E was significantly reduced in middle-aged mice compared to young mice and thereafter maintained in old mice, but there was no significant difference between young and old mice (Figure 4d). The velocity of E’ was significantly reduced in middle-aged mice compared to young mice and subsequently preserved in old mice (Figure 4e). In contrast to MV E, E’ was significantly lower in old mice than in young mice. The other parameters of diastolic function, including MV A, E/A, deceleration time (DT), isovolumic relaxation time (IVRT), IVRT/R-R interval (RR) and E/E’, are depicted in Table 2. In contrast to E/E’, we found no significant differences between young, middle and old groups. Compared to young mice, the E/E’ was significantly increased in middle-aged mice and then maintained in old mice. There was no significant difference in E/E’ between young and old mice.

### 2.5. Antioxidant Status in Young, Middle-Aged and Aged Mice

**Figure 5:**
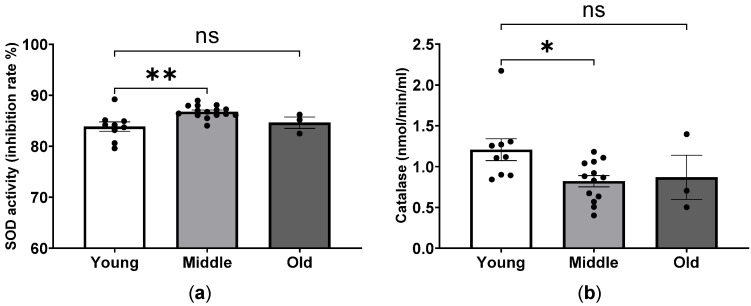
Age-dependent antioxidant capacity in mice. Total SOD (**a**) and catalase (**b**) activities in young, middle-aged and old mice. Plasma levels of SOD and catalase were measured using ELISA kits. * *p* < 0.05 and ** *p* < 0.01 between the indicated groups, ns, not significant, one-way ANOVA.

To evaluate the antioxidative capacity, plasma levels of SOD and catalase activities were measured in young, middle-aged and old mice. The mean SOD activity in young, middle, and old mice was 83.9%, 86.8%, and 84.6%, respectively. The SOD activity was significantly higher in middle-aged mice compared to young mice, whereas there were no significant differences in the SOD activity of old mice compared to middle-aged and young mice (Figure 5a). A similar reduced activity of catalase was observed in middle-aged mice as compared to the young group (Figure 5b). As shown in Figure 5b, no significant differences were found between middle-aged and old mice.

**Figure 6:**
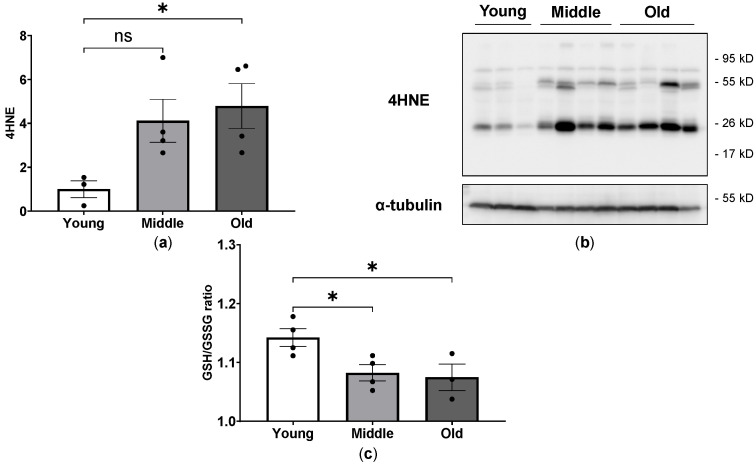
Profile of oxidative stress markers in young, middle-aged and old mice. Quantification analysis (**a**) and Western blot image (**b**) of 4-HNE in cardiac tissue from young, middle-aged and old mice. Ratio of reduced glutathione to oxidized glutathione (GSH/GSSG) in plasma from young, middle-aged and old mice (**c**). * *p* < 0.05 between the indicated groups, ns, not significant, one-way ANOVA.

We also examined the myocardial expression profile of 4-HNE, a sensitive marker of oxidative damage and lipid peroxidation, in young, middle-aged and old mice. As shown in Figure 6a,b, old mice displayed an increased level of 4-HNE in the myocardium as compared to young mice. In addition, we examined reduced (GSH) and oxidized (GSSG) glutathione levels, representing a dynamic balance between oxidants and antioxidants in mice. Compared to the young group, the ratio of GSH/GSSG was markedly reduced in middle-aged and old mice (Figure 6c).

### 2.6. Profile of Inflammatory Factors in Young, Middle-Aged and Aged Mice

**Figure 7:**
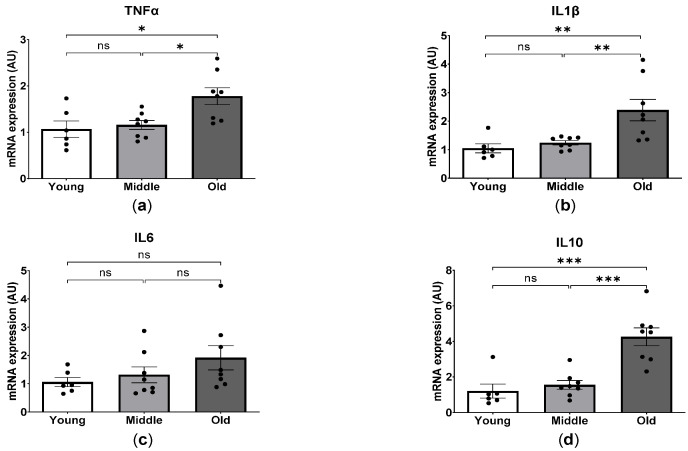
Age-dependent mRNA expression of inflammatory factors in mice. Quantitative RT-PCR analysis of mRNA expression levels of TNFα (**a**), IL1β (**b**), IL6 (**c**), and IL10 (**d**). * *p* < 0.05, ** *p* < 0.01, and *** *p* < 0.001 between the indicated groups, ns, not significant, one-way ANOVA.

Chronic inflammation plays a fundamental role in disease by favorizing the initiation and progression of age-specific cardiac abnormalities [20,21]. We next examined the myocardial expression of inflammatory cytokines in young, middle-aged and old mice. As shown in Figure 7, we found no significant differences in the myocardial expression of inflammatory factors including tumor necrosis factor-α (TNFα), interleukin-1β (IL1β), interleukin-6 (IL6) and interleukin-10 (IL10) between young and middle-aged mice. In contrast, a significantly elevated expression of TNFα, IL1β and IL10 was detected in old-aged mice compared to those in young and middle-aged mice, whereas the IL6 level remained unchanged.

**Figure 8:**
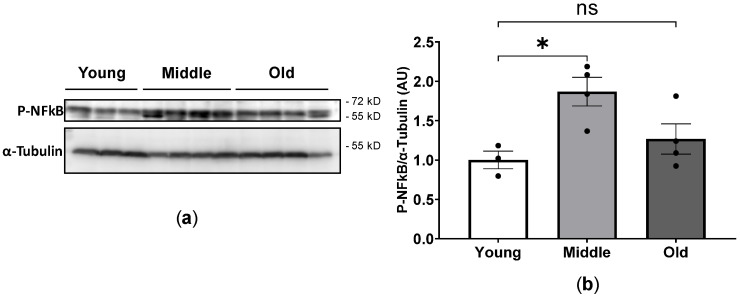
NF-κB-p65 Ser276 phosphorylation in young, middle-aged and old mice. Heart total protein extracts were resolved by SDS-PAGE and probed with antibody against p-NF-κB-p65-Ser276. Western blot image (**a**) and quantification analysis from a (**b**). * *p* < 0.05 between the indicated groups, ns, not significant, one-way ANOVA.

The transcription factor NF-κB regulates the expression of multiple proinflammatory cytokines [22,23,24]. We next analyzed levels of the phosphorylated form of NF-κB by immunoblot using whole tissue extract from young, middle-aged and old mice (Figure 8). Middle-aged mice showed increased NF-kB p65 (Ser 276) phosphorylation as compared to young mice, whereas there were no significant differences in NF-κB p65 subunit phosphorylation between young and old mice (Figure 8).

## 3. Discussion

Age-associated cardiac phenotype in the murine model closely reflects age-related functional and metabolic alterations in humans. Data from the current study demonstrate that aging progressively alters cardiac phenotype, metabolic, inflammatory and antioxidant status in mice. Elevated expression of several cytokines including IL1, IL10 and TNFα was associated with age-related progressive decline in cardiac function. While systolic activity was preserved in the middle-aged group, both EF and FS were reduced in aged mice, suggesting age-dependent progression to systolic dysfunction. Diastolic function evaluated by echocardiography also declined in aged mice as compared with young or middle-aged groups. In mice without apparent cardiovascular complications, both systolic and diastolic functions are progressively compromised with aging, suggesting that a decline in cardiac performance can predispose to the development of heart failure in aged mice. Interestingly, age-dominated impairments in cardiac function and metabolic parameters were accompanied by increased oxidative stress. These findings highlight the role of several cytokines and antioxidants in age-related events of cardiac dysfunction and may have important implications for tailored treatment of heart disease in aging.

Alterations in the structure and function of the heart accompany aging and predispose to the development and progression of heart failure [25]. Using echocardiography, the present study provides an integrated, non-invasive approach to the evaluation of systolic and diastolic cardiac function in mice, contributing to the pathophysiology of aging in the heart. Importantly, both LVID and LV mass had a progressive increase in aged mice. Previous studies have suggested that the aged C57BL/6 male mouse captures major aspects of cardiac remodeling processes that have been implicated as core pathophysiologic mediators of heart failure [9,26,27,28]. The mouse models phenocopy the human cardiovascular system. However, several distinctions between humans and mice include their different lifespans (70+ years versus 2+ years) [29,30] and body size [29]. Ventricular contraction includes cardiac output (5 L/min for humans [31], 15 mL/min for mice [32]), and heart rate (60 bpm for humans, 600 bpm for mice [33]). Nevertheless, despite the higher heart rate in mice, the number of cardiac cycles over a lifetime is still greater in humans (2.2 × 10^9^) than in mice (6.3 × 10^8^), which can impact age-associated cardiac remodeling processes [34]. Indeed, cardiac tissue aging in mice can be attributed to the accumulation of remodeled collagen and a progressive fall in structural integrity [35,36]. This reason, which distinguishes mouse models of compromised collagen fiber integrity, can be useful in cardiac aging research [35]. Further diversity between human and murine cardiac aging remains to be established. In relation to both the acquisition of aortic stiffness and ventricular remodeling, heart failure with preserved ejection fraction (HFpEF) plagues elderly patients [37]. In clinical practice, the accentuation of HFpEF in elderly patients is linked to adverse outcomes in the aging patient population, with regard to morbidity and reduced life quality [37]. To date, we have limited evidence-based interventions to stem the development of HFpEF in patients. Myocardial ischemia, due to either ischemic cardiomyopathy or subendocardial ischemia in the hypertrophied LV, can predispose to heart failure with reduced EF [37]. Thus, aging can promote the development of both major forms of heart failure, which represent a crucial challenge to the quality of life in an aging population.

Oxidative damage in the cardiovascular system may occur when antioxidant defense is perturbed to limit ROS production. Oxidant stress has been linked to the pathogenesis of age-associated cardiovascular complications and heart disease [13,38,39,40]. In this context, early observational studies centered on dietary antioxidants (β-carotene, ascorbic acid, α-tocopherol) and showed an inverse relationship between intake of these antioxidants and adverse cardiovascular events [15,41]. Importantly, these data supported a number of randomized trials of selected antioxidants as primary and secondary prevention strategies to limit cardiovascular risk; however, the majority of these studies reported disappointing results with little or no observed risk reduction in antioxidant-treated subjects [42]. Multiple explanations for these findings have been explored, including maladaptive antioxidant dose or choice, dietary versus synthetic antioxidant as the intervention, and patient selection, all of which will be important to consider for designing future clinical trials.

A shift of the prooxidant/antioxidant balance towards excessive formation of ROS leads to a wide spectrum of cardiovascular manifestations [13,14,15]. To investigate the significance of antioxidant systems in cardiac aging we measured the activities of critical antioxidant enzymes, catalase and SOD in young, middle-aged and old mice. A rise of total SOD activity which plays a role to dismutate superoxide into H_2_O_2_, was detected in middle-aged mice, while old group exhibited SOD values similar to levels found in young controls. At the same time, no differences in the activity of another antioxidant enzyme, catalase, were observed between the young, middle-aged and old groups of mice. These results attest to the predominant role of SOD in active periods of life, which can serve as the source for the maintenance of dynamic metabolism at a high level in the course of natural aging. Among the three distinct isoforms of SODs, SOD2 specifically localizes in the mitochondrial matrix [43]. Several studies have demonstrated the role of SOD2 in cardiac aging. Deletion of the SOD2 gene results in early postnatal lethality in mice [44]. Deficiency in SOD2 in mice is viable but demonstrates increased susceptibility to oxidative stress, defective mitochondrial function and elevated sensitivity to cell death [45,46]. A recent study has reported that SOD2 deficiency over a lifetime is enough to induce cardiac dysfunction [47]. In addition, SOD2 deficiency also increases collagen I in aged smooth muscle cells [48]. Further studies are required to explore the precise roles of three distinct isoforms of SOD in the course of human–animal aging.

Numerous experimental and clinical studies report that inflammatory cytokines strongly predict heart failure and multiple adverse myocardial events [20,21]. Cytokine dysregulation is believed to play a dominant role in the remodeling of the cardiac function at older age, with evidence pointing to an inadequancy to fine-control systemic inflammation reflecting unhealthy aging [49,50]. This reshaping of cytokine expression pattern, with a progressive decline in antioxidant capacity can calibrate the functional performance of the heart [38,39,40]. Chronic low-grade systemic inflammation is a well-established hallmark of aging [49,51] and is characterized by high levels of circulating cytokines in the aged population in the absence of general pathophysiological stress [49,50]. Chronic inflammation is involved in accelerated biological aging and age-related heart disease [49,52,53]. Accumulating evidence suggest an age-related increase in blood inflammatory markers including TNFα, IL1β, and IL10 [21,49,50]. This condition is known as inflammaging and a cause of accelerated aging and a comprehensive marker of multimorbidity, disability, frailty, and premature death in old adults [53,54]. In our study, we have demonstrated an age-associated increase in plasma levels of cytokines, including IL1, IL10 and TNFα, suggesting inflammaging-related cardiac remodeling. Further studies are needed to clarify the molecular age-related mechanisms leading to cellular senescence, including impaired mitochondrial function. In cardiac tissue, inflammatory cytokines are not stored intracellularly, and their secretion depends on new protein synthesis. As a consequence, the elaboration of cytokines in response to an inflammatory stimulus is significantly or predominantly regulated by the transcription rates of cytokine genes. Since transcriptional regulation is critical for the production of many cytokines, transcription factors, including NF-kB, may play key roles in regulating cytokine-mediated inflammation. In an aged heart, inflammatory and oxidative stresses stimulate the NF-κB family of transcription factors [14,52]. Therefore, as a common responder to varied stress stimuli, NF-κB is well positioned to play a capital role in driving aging. Indeed, NF-κB has been directly implicated in the aging process. For example, using motif mapping, NF-κB was determined to be the transcription factor most associated with aging [55]. Furthermore, biologic pathways implicated in aging, including immune responses, cell senescence, apoptosis and metabolism, are regulated at least in part by NF-κB. Additionally, other cellular processes implicated in regulating lifespan, including insulin/IGF-1 and growth hormone pathways, SIRT, FoxO and mTOR, are all interconnected with NF-κB signaling. Finally, the role of aberrant NF-κB signaling is well documented in numerous age-associated diseases, including neurodegeneration, osteoporosis, diabetes, sarcopenia and atherosclerosis [55].

Furthermore, the results of our study highlight the possible role of adipokine resistin, in age-related cardiac dysfunction. Interestingly, we found that age-mediated changes in metabolic parameters associated with cardiovascular risk in mice were accompanied by elevated levels of resistin. Alterations in resistin levels in aged mice depend on the metabolic parameters of the animals [56,57]. In humans, resistin levels are linked to the metabolic and cardiovascular parameters in cases of metabolic disorders [54,56]. Resistin demonstrates its inflammatory role by intracellular activation of NF-kB, which calibrates the trajectories of inflammatory cytokine secretion [57,58]. An extensive study is needed to establish correlations between resistin and antioxidant capacity in aged hearts.

### Limitations of the Study

Our study has several limitations. We did not execute a longitudinal study design to explore trajectories of cardiac function decline with age in relation to the inflammatory and oxidant/antioxidant status. Rather, we performed a cross-sectional study design that compared different ages of mice to examine age-specific cardiac and metabolic phenotypes. While longitudinal analyses have their strengths (i.e., repeated measurements on the same animal), they are certainly not without obstacles and have their own sets of limitations including in vivo complications of performing assays and potential effects on the outcome of subsequent repeated tests. Another critical issue linked to longitudinal designs is that measurements cannot be taken at the same time but instead require a comparison of data collected at different time points, corresponding to 2 years apart in our study. Therefore, longitudinal studies may not naturally generate more robust values of age-related trajectories in mouse populations than population estimates derived from cross-sectional data collected at the same time and under the same well-controlled conditions. According to these considerations, it is common practice to explore aging-associated cardiac phenotypes in studies using cross-sectional designs. An additional critical point is that we did not standardize LV mass to BW in mice since there is controversy regarding the optimal method for indexing LV mass to body BW in the clinical setting. Indeed, the prediction of values of LV by BW is more accurate at birth and progressively less precise with increasing age. Moreover, in this work, we did not take into consideration the sex-dependent differences in cardiac phenotype in mice; in fact, there is growing experimental and clinical evidence supporting the existence of sex-specific patterns of cardiac aging [59]. Thus, these aspects need to be further investigated.

## 4. Materials and Methods

### 4.1. Animals

All animal work described in this investigation was performed in accordance with institutional guidelines for animal research and were approved by the French Ministry of Research in agreement with European Union guidelines according to the Directive 2010/063 EU [60] and French Decree 2013-118 [61]. C57BL/6 male mice aged 3 months (young group, n = 11), 12 months (middle-aged group, n = 15) and 24 months (old group, n = 4) (Envigo RMS, Gannat, France) were used for the echocardiographic measurements of cardiac structure and function. Mice were housed in groups of five in polycarbonate cages, enriched with paper and were allowed free access to standard food and water.

### 4.2. Echocardiography

Mice were anesthetized with isoflurane and underwent echocardiography in the supine position using a Vevo 2100 high-frequency high-resolution ultrasound System (VisualSonics, Toronto, ON, Canada). Electrocardiogram (ECG) and heart rate (HR) were monitored using limb electrodes. Body temperature was measured using a rectal probe and maintained at 37 °C. Chest hair was removed with a depilatory cream or a shaver. The parasternal long-axis imaging, the parasternal short-axis B-mode and M-mode imaging, and the apical four-chamber imaging were recorded. Left ventricular end-diastolic (LVEDV) and end-systolic volume (LVESV), SV, CO, and EF were obtained from the parasternal long-axis image by tracing the left ventricle in diastole and systole. FS, LVID in diastole (LVIDd) and in systole (LVIDs), LVAW in diastole (LVAWd) and in systole (LVAWs), LVPW in diastole (LVPWd) and in systole (LVPWs), and LV mass were obtained from the parasternal short-axis M-mode image. The MV inflow velocity was acquired using the pulsed-wave Doppler and placing the sample volume at the tip of the mitral valve leaflet in the apical four-chamber view. Mitral inflow E wave velocity (E), mitral inflow A wave velocity (A), E velocity DT, IVRT, and isovolumic contraction time (IVCT) were measured from the mitral valve inflow imaging. The tissue Doppler imaging was acquired with the sample volume placed on the septal mitral valve annulus in the apical four-chamber view. The tissue Doppler-derived septal mitral annular E’ (E’) and A’ wave velocity (A’) were obtained from the tissue Doppler imaging.

### 4.3. Metabolic Parameters

To determine the amount of body fat mass, lean mass, and free body fluid weight we used a nuclear magnetic resonance (NMR) based instrument (Minispec, Bruker, MA, USA). This approach provides the measurements non-invasively and without the need for anesthesia.

### 4.4. Tail-Tip Whole Blood Sampling

Whole blood was collected from the tail vein of conscious mice using EDTA-coated microvettes up to a maximum volume of 100–150 μL. The samples were then centrifuged at 3800 rpm for phase separation, and the plasma was frozen and stored at −80 °C for future assays. After collection, the bleeding was stopped by applying slight pressure on the incision using a cotton pad. Catalase (707002, Cayman Chemical Company, Ann Arbor, MI, USA), SOD (19160, SIGMA Life Science, Buchs, Switzerland) and resistin (A05178, SPIbio, Montigny le Bretonneux, France) levels were measured using a commercially available ELISA kits according to the manufacturer’s protocol.

### 4.5. Immunoblotting Analysis 

For immunoblot analyses, heart tissues were lysed in RIPA buffer (89900, Pierce, Appleton, WI, USA) supplemented with protease inhibitor cocktail (P8340, Sigma-Aldrich, St. Louis, MO, USA) and Halt Phosphatase inhibitor cocktail (78426, Thermo Scientific, Waltham, MA, USA). Protein concentrations were determined by BCA protein assay reagent (23225, Thermo Scientific). Protein extracts were separated on 12% SDS–PAGE gels and transferred to PVDF membranes (IPVH00010, Millipore, Burlington, MA, USA). Membranes were incubated with the primary antibodies anti-4-Hydroxynonenal, (MA5-27570, Invitrogen, Carlsbad, CA, USA), anti-phospho-NF-kB, (ab106129, Abcam, Cambridge, UK) or α-Tubulin (T6074, Sigma-Aldrich) overnight at 4 °C, then for 2 h with HRP-conjugated secondary antibodies (Cell Signaling Technologies, Danvers, MA, USA). Membranes were revealed by chemiluminescence using Clarity Western ECL substrate (170-5060, BioRad, Hercules, CA, USA) on a Chemidoc Touch (BioRad).

### 4.6. GSH/GSSG Ratio Assay

The ratio of reduced (GSH) to oxidized glutathione (GSSG) was determined in young, middle-aged and old mice plasma using the GSH/GSSG Ratio Detection Assay Kit (ab138881, Abcam) according to manufacturer.

### 4.7. Quantitative RT–PCR Analysis

The expression of genes was assessed using quantitative polymerase chain reaction (qRT-PCR). Total RNAs were isolated from cardiac tissue using the RNeasy mini kit (Qiagen, Hilden, Germany). The sequences of the primers used are as follow and were given in the 5′-3′ orientation: TNFa ATCTCATACCAGGAGAAAGTCAACCT (forward) and ATGGGCTCATACCAGGGTTTG (reverse); IL 1β CTGCACTACAGGCTCCGAGAT (forward) and TGTTGGTTGATATTCTGTCCATTGA (reverse); IL 6 AAGTGCATCGTTGTTCATACA (forward) and GAGGATACCACTCCCAACAGACC (reverse); IL-10 TGAGGCGCTGTCATCGATTT (forward) and TGGCCTTGTAGACACCTTGG (reverse); resistin TCATTTCCCCTCCTTTTCCTTT(forward) and TGGGACACAGTGGCATGCT (reverse); 36b4 GCT-TCA-TTG-TGG-GAG-CAG-AC (forward) and ATG-GTG-TTC-TTG-CCC-ATC-AG (reverse). The expression of target gene was normalized to 36b4 housekeeping gene expression.

### 4.8. Statistical Analysis

Continuous variables were expressed as the mean ± SEM. A one-way analysis of variance (ANOVA) followed by Tukey’s multiple comparisons test for multiple pairwise comparisons was performed using GraphPad Prism software (version 9.5.0, GraphPad Software, Inc., La Jolla, CA, USA). A *p* value of less than 0.05 was considered to be statistically significant.

## 5. Conclusions

In conclusion, this study demonstrated that age-dependent trajectories of cardiac function decline are associated with dynamic changes in oxidative stress and antioxidant status markers, as well as metabolic and cytokine dysregulation. We show that in mice without apparent cardiovascular manifestations, both systolic and diastolic functions are progressively compromised with age, suggesting that a decline in cardiac activity can predispose to the development of heart failure in aged mice. Age-specific differences in cardiac phenotype linked to the inflammatory and antioxidant status in middle- and aged animals may have important implications for tailored treatment of cardiovascular defects in aging.

## Figures and Tables

**Table 1:** The metabolic characteristics and their comparisons by age in C57BL/6 mice.

Parameters	Young	Middle	Old
BW (g)	33.18 ± 1.00	46.20 ± 1.51 ****	34.33 ± 3.84 §§
Fat (g)	4.18 ± 0.80	12.86 ± 0.82 ****	4.00 ± 1.98 §§§
Lean tissue (g)	23.65 ± 0.45	25.73 ± 0.56 *	23.91 ± 1.94
Free body fluid (g)	0.92 ± 0.05	1.36 ± 0.06	2.91 ± 1.56 **§

All values are represented as mean ± SEM. BW, body weight; HR. * *p* < 0.05, ** *p* < 0.01, and **** *p* < 0.0001 vs. young mice. § *p* < 0.05, §§ *p* < 0.01, and §§§ *p* < 0.001 vs. middle-aged mice.

**Table 2:** Echocardiographic parameters in young, middle-aged and aged mice.

Parameters	Young	Middle	Old
HR (bpm)	472.7 ± 20.56	456.0 ± 11.52	455.5 ± 30.73
SV (µL)	36.63 ± 0.91	38.49 ± 1.14	30.45 ± 3.21 *§§
CO (mL/min)	17.17 ± 0.50	17.45 ± 0.65	13.93 ± 1.31 §
LVEDV (µL)	64.41 ± 0.95	66.63 ± 2.00	73.78 ± 3.57 *
LVESV (µL)	27.78 ± 0.79	28.14 ± 1.27	43.32 ± 0.96 ****§§§§
LVAWd (mm)	0.67 ± 0.01	0.75 ± 0.02 **	0.65 ± 0.04 §§
LVAWs (mm)	1.03 ± 0.03	1.09 ± 0.02	0.88 ± 0.03 *§§§
LVPWd (mm)	0.71 ± 0.02	0.80 ± 0.02 ***	0.72 ± 0.01 §
LVPWs (mm)	1.00 ± 0.02	1.15 ± 0.02 ***	1.01 ± 0.02 §
A (mm/s)	469.8 ± 17.7	430.4 ± 31.3	426.4 ± 60.4
E/A	1.49 ± 0.06	1.42 ± 0.07	1.39 ± 0.11
DT (ms)	19.27 ± 1.31	19.87 ± 1.67	20.74 ± 2.09
IVRT (ms)	17.35 ± 0.62	15.88 ± 0.96	20.00 ± 1.53
IVRT/RR	0.13 ± 0.01	0.12 ± 0.01	0.14 ± 0.004
E/E’	21.71 ± 1.49	31.71 ± 2.56 *	31.79 ± 4.65

All values are represented as mean ± SEM. A, mitral inflow A wave velocity; CO, cardiac output; DT, E velocity deceleration time; E, mitral inflow E wave velocity; E’, tissue Doppler-derived septal mitral annular E’ wave velocity; HR, heart rate; IVRT, isovolumic relaxation time; LVAWd, left ventricular anterior wall thickness in diastole; LVAWs, left ventricular anterior wall thickness in systole; LVEDV, left ventricular end-diastolic volume; LVESV, left ventricular end-systolic volume; LVPWd, left ventricular posterior wall thickness in diastole; LVPWs, left ventricular posterior wall thickness in systole; RR, R-R interval; SV, stroke volume. * *p* < 0.05, ** *p* < 0.01, *** *p* < 0.001, and **** *p* < 0.0001 vs. young mice. § *p* < 0.05, §§ *p* < 0.01, §§§ *p* < 0.001, and §§§§ *p* < 0.0001 vs. middle-aged mice.
